# *Pseudomonas aeruginosa* infection causes lysosomal dysfunction in the cystic fibrosis bronchial epithelium

**DOI:** 10.1128/iai.00347-26

**Published:** 2026-06-30

**Authors:** Emma Lea Matthews, Meghan June Hirsch, Kuen-You Tsai, Hannah J. McIntire-Ray, Viviana Acevedo Rua, Angela N. Morales, Luke I. Jones, Jarrod W. Barnes, Megan R. Kiedrowski, Stefanie Krick

**Affiliations:** 1 Division of Pulmonary, Allergy & Critical Care Medicine, Heersink School of Medicine, The University of Alabama at Birmingham 654677, Birmingham, Alabama, USA; University of Pennsylvania Perelman School of Medicine, Philadelphia, Pennsylvania, USA

**Keywords:** lysosome, cellular senescence, bronchial epithelium, pulmonary infection, *Pseudomonas aeruginosa*, cystic fibrosis

## Abstract

Cystic fibrosis (CF) is a genetic disorder that causes chronic airway disease, leading to accelerated aging, inflammation, and susceptibility to opportunistic infections. *Pseudomonas aeruginosa* (PA) is prevalent in the CF lung, often multidrug-resistant, and affecting lung function decline, highlighting a need for alternative treatment strategies, such as host-directed therapeutics. The PA metabolite pyocyanin can induce senescence-associated β-galactosidase (SA-βgal), a lysosomal cell senescence marker. However, whether PA can induce cell senescence and therefore affect immune functions has not been studied. Using established airway epithelial culture models, we observed that PA infection increased SA-βgal activity and proinflammatory cytokines without increasing a comprehensive set of cell senescence markers. PA infection also resulted in lysosomal neutralization and decreased lysosome biogenesis, with the latter attenuated by rapamycin (RAPA), a senolytic and an established inhibitor of mTOR signaling. In conclusion, our study implies that PA infection does not induce cell senescence in the CF bronchial epithelium but disturbs lysosomal homeostasis, which can be attenuated by RAPA. These findings highlight lysosomal health as a promising therapeutic target in CF airways, especially as patients live longer yet remain susceptible to recurrent airway infections.

## INTRODUCTION

Cystic fibrosis (CF) (1) is a genetic disorder that affects over 105,000 people globally (2). CF is caused by a mutation in the cystic fibrosis transmembrane conductance regulator (CFTR) gene, leading to a defective CFTR protein and resulting in a multisystem disorder due to its wide expression by epithelial surfaces. One of the most prominent and pathological manifestations is airway disease, which is also the most common cause of CF-related morbidity and mortality (2–4). CFTR dysfunction leads to thickened mucus and remodeling of the airway epithelium, creating an environment for opportunistic bacteria such as *Pseudomonas aeruginosa* (PA) to colonize and establish recurrent infections (3, 5). PA is a gram-negative bacterium that is normally found in the environment and is the most prevalent cause of chronic respiratory infections in adult people with CF (pwCF) (2, 6). Despite the wide use of highly effective modulator therapy (HEMT), which improves CFTR function, PA colonization can persist in pwCF, especially in those with pre-existent lung damage (2, 7). This is especially true for the mucoid phenotype of PA, which overproduces the exopolysaccharide alginate that aids PA in producing biofilms (8). Biofilm production by PA can increase its resistance to antibiotic treatment, and colonization with mucoid-PA is associated with worse lung function and increased mortality in CF patients (9). It is, therefore, important to continue developing additional therapies to treat recurrent infections.

Since HEMT has led to significant improvements in lung function, thereby prolonging the life expectancy of most pwCF, aging itself has become a focus in CF care and the promotion of healthy aging to improve the quality of life (10). Hallmarks of aging at the tissue and cellular levels have been identified in CF, including cellular senescence, defined by irreversible cell cycle growth arrest, apoptotic resistance, and an increase in proinflammatory markers, which occurs in response to cellular stressors such as inflammation (11, 12). Senescent cells are resistant to apoptosis and acquire a senescence-associated secretory phenotype (SASP), which stimulates the secretion of proinflammatory mediators that perpetuate tissue damage and chronic inflammation (13). Biomarkers of senescence include B-cell lymphoma-extra-large (BCL-xL), indicating apoptotic resistance; cyclin-dependent kinase inhibitor 2A (p16) and cyclin-dependent kinase inhibitor 1 (p21) for cell cycle growth arrest; interleukin-1β (IL-1β), IL-6, and IL-8 for SASP; and increased activity of senescence-associated β-galactosidase (SA-βgal), a marker of increased lysosomal content from continued cellular metabolic activity without apoptosis (12, 14–16). Previously, we have shown that accelerated aging is observed in the CF bronchial epithelium through an increase in the cellular senescence markers and inflammatory SASP (17). The PA metabolite pyocyanin (PCN) has been observed to increase SA-βgal activity by triggering reactive oxygen species (ROS) production (18, 19), but to date, no study has comprehensively assessed the impact of PA infection on cellular senescence in CF.

Cellular senescence has been shown to be regulated by the autophagy-lysosome degradation pathway (20). Lysosomes are acidic, intracellular organelles that are responsible for degrading cellular waste, including through autophagy, making them central to regulating cellular stress responses (21, 22). Studies have shown that PA can gain access to phagocytic cells such as macrophages to disrupt autophagy pathways (23). However, only a few studies have investigated how PA can disrupt lysosomal homeostasis in lung epithelial cells, a crucial barrier to defend against infection (24, 25). PA can sometimes act as an intracellular pathogen (26), can be cleared by epithelial cells via lysosomal degradation (25), and has been shown to downregulate lysosomal/autophagic machinery (24). While studies have shown that CFTR defects do not intrinsically change lysosomal pH (27, 28), no studies have explored how PA disrupts lysosomal homeostasis in the CF lung.

Given the previously stated evidence that the CF bronchial epithelium shows an increase in cell senescence and PA causes inflammation in the CF airways, we investigated whether PA can further increase cell senescence after prolonged infection of the CF airway epithelium and whether PA affects SA-βgal and the lysosomal machinery in this accelerated aging process.

## RESULTS

### Pyocyanin increased SA-βgal activity but did not change the expression levels of other cell senescence markers in CF bronchial epithelial cells

**Fig 1:**
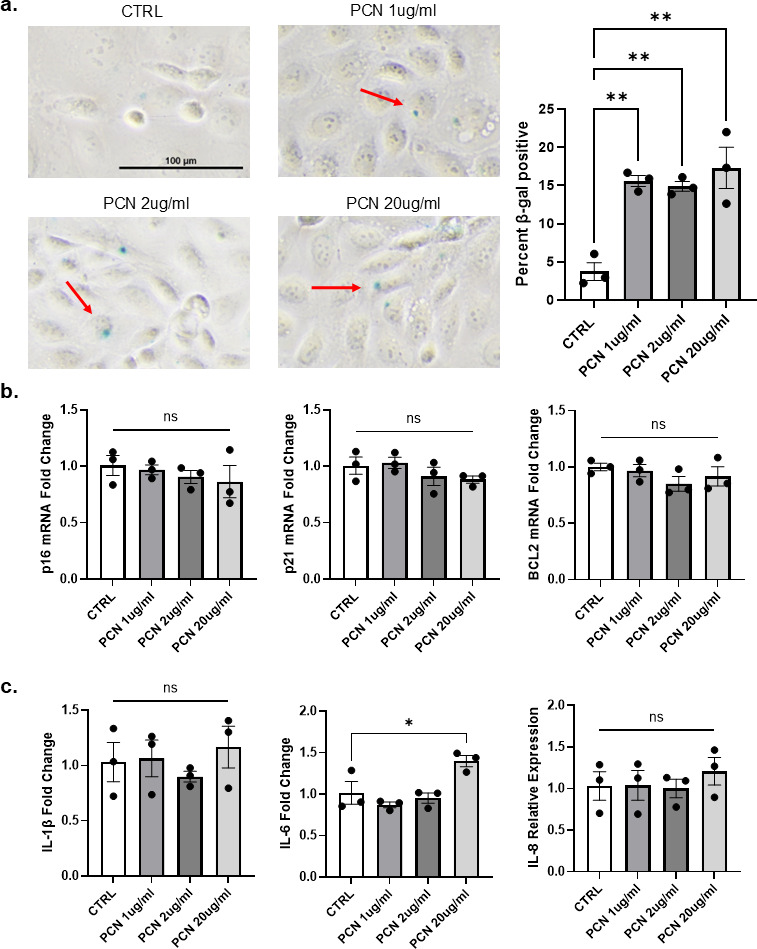
PCN treatment upregulates SA-βgal activity independently of other senescence markers in CFBEs. (**a**) Representative images for SA-βgal staining and quantification in CFBEs after 48-h PCN 1 µg/mL, 2 µg/mL, or 20 µg/mL treatment; red arrows indicate examples of SA-βgal-positive cells; quantification was completed using the ratio of SA-βgal-positive cells over total cells in each brightfield image by ImageJ. (**b**) Fold change of mRNA transcript levels of cellular senescence markers *p16, p21,* and *BCL2* and (**c**) proinflammatory cytokines *IL-1β, IL-6,* and *IL-8* in CFBEs after 48-h PCN treatment. All values shown are mean ± SEM with *P ≤ 0.05, **P < 0.01, and ***P < 0.001. Statistical significance was analyzed via unpaired Student’s *t*-test (*N* = 3).

In order to recapitulate previous findings that the PA metabolite PCN could increase SA-βgal activity, which was postulated as an increase in cellular senescence (18), we treated submerged CFBEs for 48 h with 1 µg/mL, 2 µg/mL, and 20 µg/mL PCN (Fig. 1). These concentrations were chosen based on average PCN concentrations found in the CF lung (29). We found that all three concentrations significantly upregulated SA-βgal positivity (Fig. 1a), as previously reported; however, none of the concentrations significantly increased additional markers representative of cell senescence, including cell cycle arrest markers p16 and p21 and anti-apoptotic marker BCL-xL (Fig. 1b). The SASP marker IL-6 was only increased at the highest concentration of PCN (Fig. 1c). These specific molecular markers of cellular senescence are unchanged with treatments, leading us to conclude that the PA metabolite, PCN, increased SA-βgal activity without affecting other cell senescence markers.

### PA infection of bronchial epithelial cells increased SA-βgal activity without increasing other cell senescence markers

**Fig 2:**
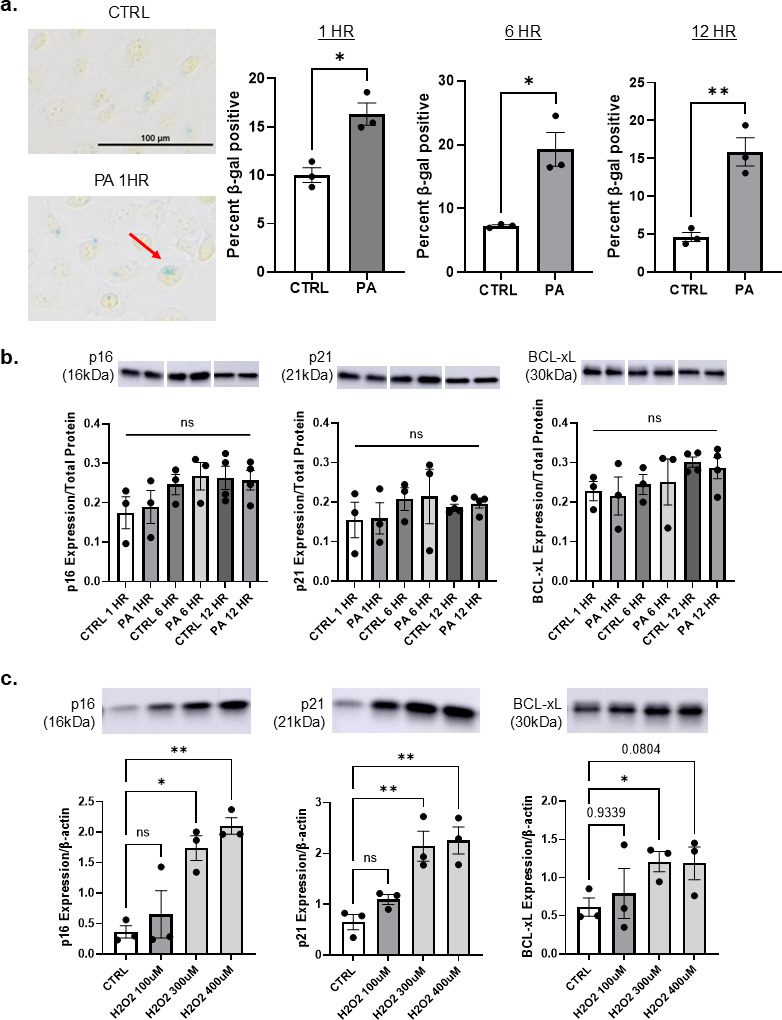
PA infection upregulates SA-βgal activity independently of other senescence markers. (**a**) Representative images for SA-βgal staining in CFBEs after 1-h PA infection and quantification in CFBEs after 1-, 6-, or 12-h PA infection; red arrows indicate examples of SA-βgal-positive cells; quantification was completed using the ratio of SA-βgal-positive cells over total cells in each brightfield image by ImageJ. (**b**) Representative immunoblot images and bars indicating quantification via densitometry of p16, p21, and BCL-xL protein levels in CFBEs after 1-, 6-, or 12-h PA infection or (**c**) 24-h H_2_O_2_ treatment; Coomassie total protein staining or β-actin was used as loading controls. All values shown are expressed as means ± SEM with *P ≤ 0.05, **P < 0.01, and ***P < 0.001. Original western blots presented are available in Fig. S1. Statistical significance was analyzed via one-way analysis of variance (ANOVA) followed by Tukey’s multiple comparison *post hoc* test (*N* = 3–4).

**Fig 3:**
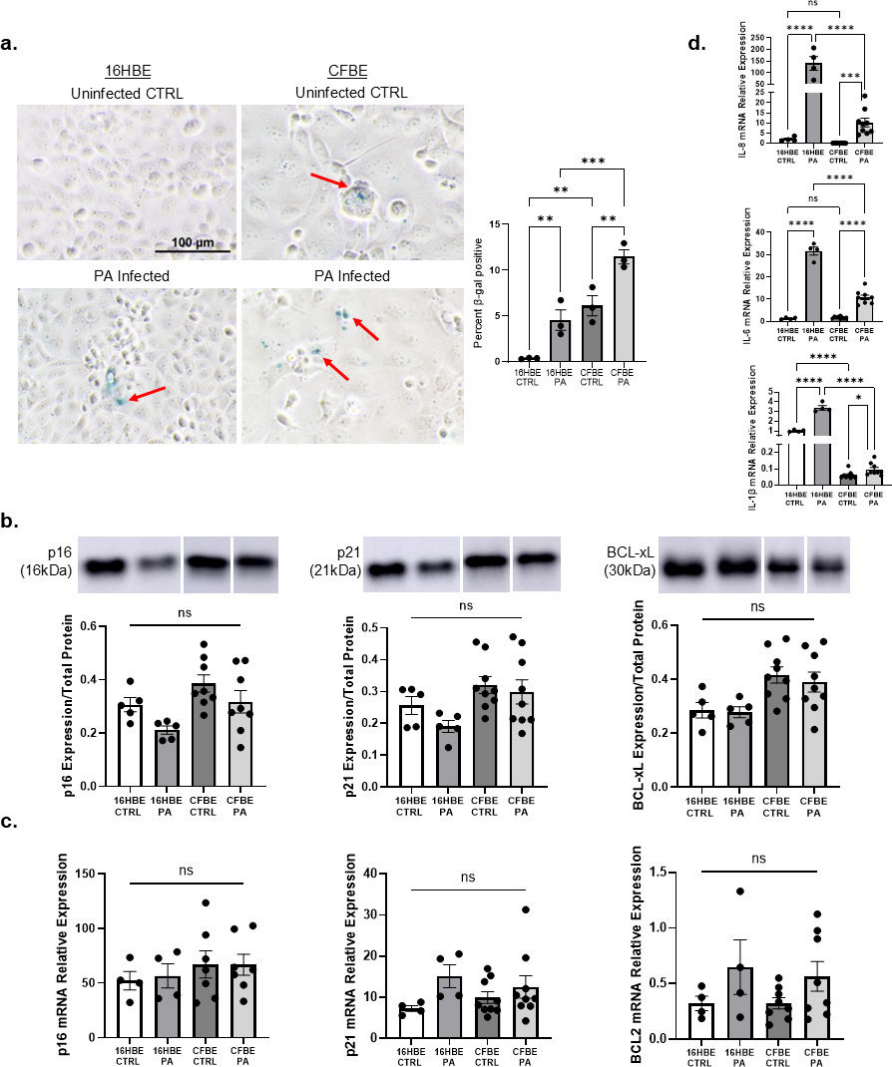
Prolonged PA infection upregulates SA-βgal activity independently of other senescence markers in both WT and CFBEs. (**a**) Representative images for SA-βgal staining and quantification in 16HBEs and CFBEs after 24-h PA infection; red arrows indicate examples of SA-βgal-positive cells (*N* = 3). (**b**) Representative immunoblot images, densitometric analyses, and (**c**) fold change of mRNA levels of p16, p21, and BCL-xL in 16HBEs and CFBEs after 24-h PA infection; Coomassie staining was used as a loading control for immunoblot quantification (*N* = 5 or 9). (**d**) Fold change of mRNA levels of *IL-1β, IL-6,* and *IL-8* in 16HBEs and CFBEs after 24-h PA infection (*N* = 4–9). All values shown are expressed as means ± SEM with *P ≤ 0.05, **P < 0.01, and ***P < 0.001. Original western blots presented are available in Fig. S2. Statistical significance was analyzed via two-way ANOVA followed by Tukey’s multiple comparison *post hoc* test.

For closer mimicking of the chronically infected CF airway, we moved it into our previously established *in vitro* PA infection model (30). We utilized a clinical mucoid PA strain that was isolated from a CF patient, PAM57-15. CFBEs were infected with PA for 1, 6, and 12 h (Fig. 2). Although SA-βgal activity was increased at each time point tested (Fig. 2a), no significant changes at the protein level of the cellular senescence markers p16, p21, and BCL-xL were detected (Fig. 2b). As a positive control of these senescence markers, we treated CFBEs with different levels of hydrogen peroxide (H_2_O_2_) for 24 h (31), and found that increasing H_2_O_2_ treatment upregulated p16, p21, and BCL-xL in our CFBE model (Fig. 2c). To ensure that we captured the effect of prolonged infection *in vitro*, we extended our infection to a 24-h model (30) and compared CFBEs with wild-type 16HBEs (Fig. 3). Consistent with the earlier time points and previous literature (17), SA-βgal remained increased after a 24-h PA infection in both CFBEs and 16HBEs and was further exacerbated in the CFBE groups (Fig. 3a). No significant changes at the mRNA or protein levels of cellular senescence markers p16, p21, and BCL-xL were detected in either cell type when infected with PA (Fig. 3b and c), except for increases in the proinflammatory cytokines IL-1β, IL-6, and IL-8 (Fig. 3d), which were attenuated in CFBEs. After considering multiple PA infection time points and non-CF cell-type specificity, the markers of senescence remain unchanged.

### Cellular senescence markers were validated using scoring of RNA sequencing data from primary, differentiated CFBEs infected with PA

**Fig 4:**
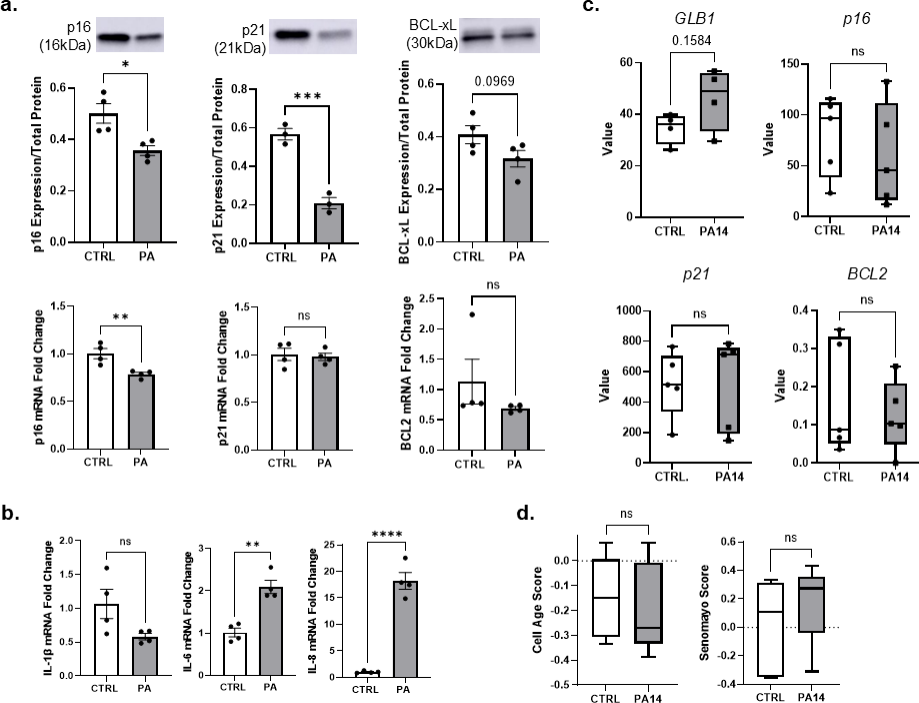
Senescence markers are not upregulated by PA in other models of CFBEs. (**a**) Representative immunoblot images, densitometric quantifications, and fold change of mRNA levels of p16, p21, and BCL-xL in CFBEs differentiated at the ALI after 24-h PA infection (*N* = 3–4). (**b**) Fold change of mRNA levels of *IL-1β, IL-6*, and *IL-8* in ALI CF cultures after 24-h PA infection (*N* = 4). (**c**) Bulk RNA sequencing values of *GLB1, p16, p21,* and *BCL2* from primary, differentiated CFBEs from five donors (*N* = 5) after 6-h PA14 infection. (**d**) Senescence scores generated from CellAge and SenMayo gene sets compared to bulk RNA sequencing from primary, differentiated CFBEs after 6-h PA14 infection. Original western blots presented are available in Fig. S3. Statistical analysis was done using unpaired Student’s t test with *P ≤ 0.05, **P < 0.01, and ***P < 0.001.

In order to validate our results in Fig. 3 in more advanced *in vitro* models of CFBEs, we repeated our 24-h PA infections using different CFBEs (CFBE41o-) with the same CFTR mutation that had been polarized at the air-liquid interface (ALI) (Fig. 4; Fig. S4) to more accurately mimic the physiology of epithelial cells lining the airway (17, 30, 32). In this model, PA infection led to a decrease in p16 and p21 protein levels and no changes in BCL-xL (Fig. 4a). Proinflammatory markers were increased after PA infection, as consistent with previous results and our own data (Fig. 4b). We wanted to take this into another advanced *in vitro* model of CFBEs derived from CF patients and cultured at the ALI using a published bulk RNA sequencing (RNA-seq) data set. We mined this published transcriptomic data set that was generated using primary, differentiated CFBEs that were infected with a virulent PA strain (PA14) for 6 h. Re-analysis of this data set revealed that p16, p21, and BCL2 levels were unchanged, but that the level of GLB1, the gene responsible for β-galactosidase transcription, increased with PA infection (Fig. 4c). To further validate these results with a more comprehensive set of cellular senescence markers, we used the published CellAge and SenMayo gene sets of markers known to be associated with aging and cellular senescence to generate comparative senescence scores, as previously described (17, 33, 34). We found that there was no significant change in the senescence scores of uninfected primary CFBEs when compared to those infected with PA14 (Fig. 4d). Taken together, these data indicate that PA leads to an increase in SA-βgal, a lysosomal cell senescence marker, but does not change other markers of cell senescence for cell cycle arrest and apoptotic resistance.

### PA infection neutralizes lysosomal pH of CFBEs

Given that SA-βgal activity is a measurement of lysosomal β-galactosidase activity at a neutral pH of 6.0, we wanted to assess the effect of PA infection on lysosomal homeostasis and decided to focus on pH regulation in PA-infected CFBEs. This is because SA-βgal activity is distinct from β-galactosidase activity as it is measured at a more neutral pH of 6 (12). We used Lysosensor Yellow/Blue conjugated to dextran as a pH indicator in CFBEs in conjunction with PA treatments (Fig. S1). The lysosome should be at acidic pH ~4.5, but we observed a substantial decrease in the acidic/neutral ratio of PA-infected CFBEs (Fig. S5a). Once this ratio was plotted against a standard curve of known pH measurements to acidic/neutral emission ratios (Fig. S5c), we also observed a positive shift in pH (Fig. S5b). These data indicate that PA infection can neutralize endosomal and/or lysosomal pH.

### PA infection causes lysosomal dysregulation that is partially regulated by rapamycin and mTOR

**Fig 5:**
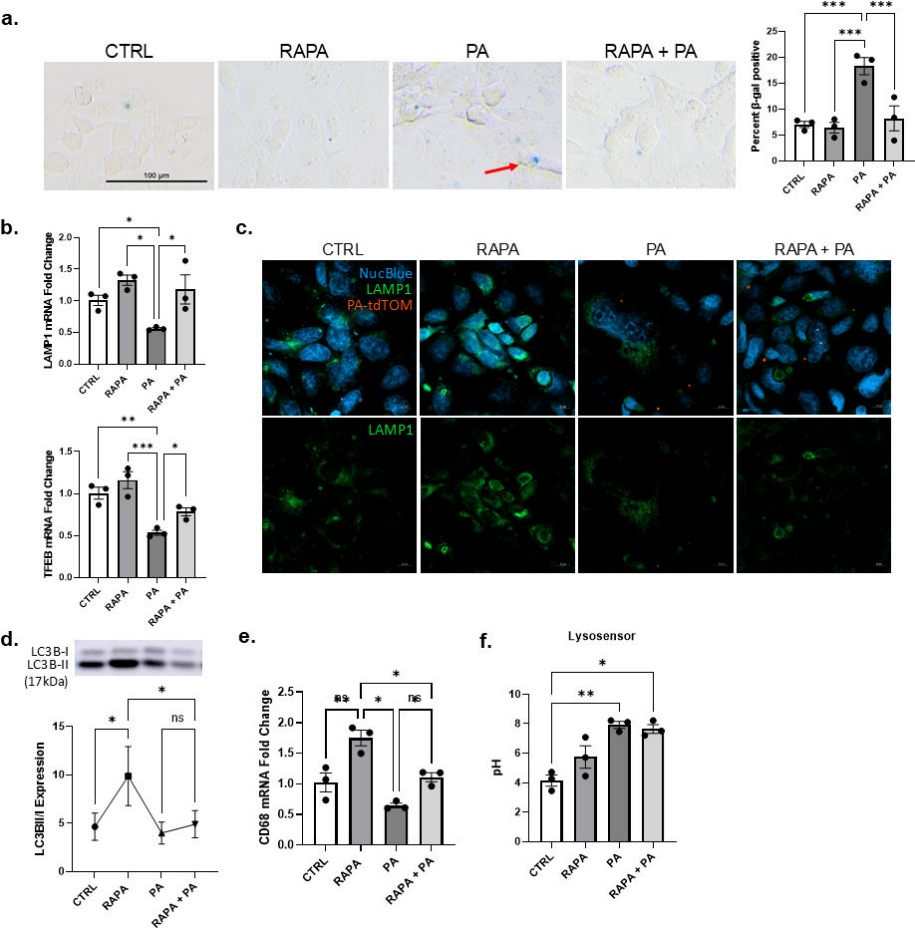
RAPA pretreatment attenuates PA-induced SA-βgal activity and lysosomal biogenesis. (**a**) Representative images for SA-βgal staining and quantification in CFBEs after 1-h RAPA pretreatment and 24-h PA infection; red arrows indicate examples of SA-βgal-positive cells; quantification was completed using the ratio of SA-βgal-positive cells over total cells in each brightfield image by ImageJ. (**b**) Fold change of mRNA levels of *LAMP1* and *TFEB* quantification in CFBEs after 1-h RAPA pretreatment and 24-h PA infection. (**c**) Immunofluorescence staining of CFBEs after 1-h RAPA pretreatment and 24-h PA infection; blue represents NucBlue, green represents LAMP1, and red represents TdTomato-PA. (**d**) Representative immunoblot image and densitometric quantification of LC3BI and LC3BII, reported as LC3BII/I, in CFBEs after 1-h RAPA pretreatment and 24-h PA infection; Coomassie total protein staining was used as a loading control for immunoblot quantification. (**e**) Fold change of mRNA levels of *CD68* quantification in CFBEs after 1-h RAPA pretreatment and 24-h PA infection. (**f**) pH quantified using Lysosensor in CFBEs after 1-h RAPA pretreatment and 24-h PA infection. All values shown are expressed as means ± SEM with *P ≤ 0.05, **P < 0.01, and ***P < 0.001. Original western blots presented are available in Fig. S6. Statistical significance was analyzed via two-way ANOVA followed by Tukey’s multiple comparison *post hoc* test (All *N* = 3).

SA-βgal activity is linked to lysosomal function, which can be tightly regulated by mTOR signaling. To explore pathways that regulate SA-βgal activity and lysosomal function, we used an mTOR inhibitor and senolytic agent, rapamycin (RAPA) (35, 36) (Fig. 5). Pretreatment with 100 nM RAPA for 1 h and infection with PA for an additional 23 h revealed an attenuation of the PA-induced increase in SA-βgal activity (Fig. 5a). To further explore lysosomal-related consequences with PA infection, we measured the lysosomal biogenesis marker transcripts *LAMP1* and *TFEB* (37, 38) and found that PA markedly decreased *LAMP1* and *TFEB* expression, which was attenuated by RAPA pretreatment (Fig. 5b). To investigate spatially how PA and mTOR inhibition affected the lysosome, immunofluorescence staining was used to show LAMP1 protein in PA-infected CFBEs without PA and LAMP1 colocalization (Fig. 5c), indicating that PA may be acting on them indirectly.

To measure the functional outcomes of lysosomal dysregulation, we measured the aspects of three processes that lysosomes are essential for: autophagy, phagocytosis, and lysosome pH. We found that RAPA treatment increased the ratio of LC3BII/I, indicating an increase in lysosomal turnover via autophagy, as previously described (39). However, PA infection prevented the RAPA-induced increase in the ratio of LC3BII/I, indicating that PA affects autophagy regulation (Fig. 5d). We also found that PA infection prevented a RAPA-induced increase in the expression of CD68, which is normally a marker for macrophages but has been implicated in epithelial cell responses to bacteria (40, 41). Specifically, CD68 in epithelial cells has been hypothesized to enable a macrophage-like response and increase phagocytosis using lysosomal machinery. We found that PA decreases CD68 and RAPA treatment mitigates the CD68 decrease, indicating PA-induced CD68 changes can be changed by RAPA treatment (Fig. 5e). Additionally, we found that RAPA pretreatment could not restore lysosomal pH in our model (Fig. 5f). These data indicate that PA can disrupt lysosomal homeostasis, and the interference of these markers with RAPA treatment suggests that PA infection affects lysosomes along the mTOR axis.

### Rapamycin increased the PA-induced proinflammatory response in CFBEs independently of reactive oxygen species

**Fig 6:**
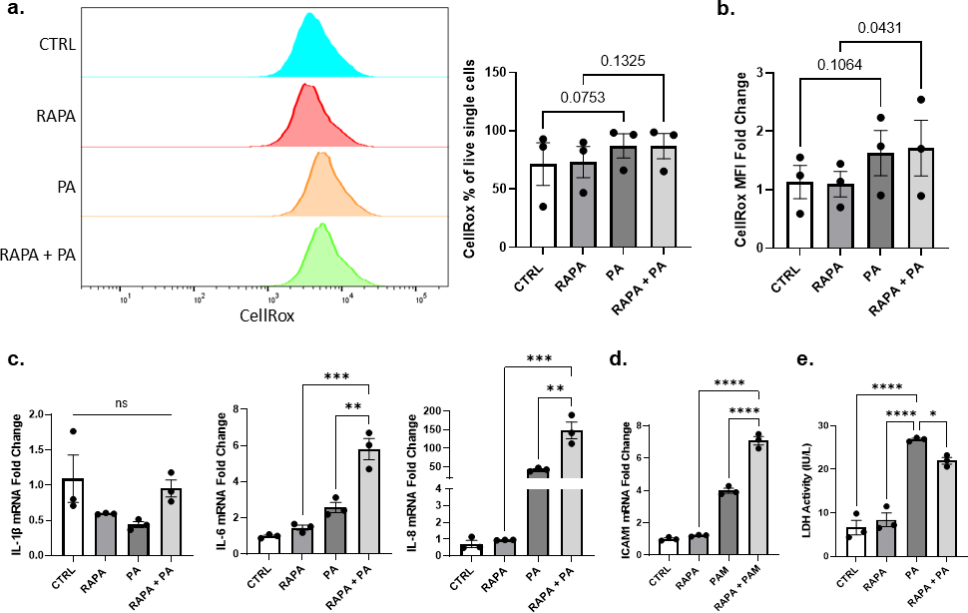
Rapamycin can increase the PA-induced proinflammatory response independently of ROS generation. (**a**) Histogram and percent CellRox positive of live, single cells and (**b**) CellRox MFI fold change in CFBEs after 1-h RAPA pretreatment and 24-h PA infection. (**c**) Fold change of mRNA levels of *IL-1β, IL-6*, and *IL-8* in CFBEs after 1-h RAPA pretreatment and 24-h PA infection. (**d**) Fold change of mRNA levels of *ICAM1* in CFBEs after 1-h RAPA pretreatment and 24-h PA infection. (**e**) LDH activity in international units per liter (IU/L) in CFBEs after 1-h RAPA pretreatment and 24-h PA infection. All values shown are expressed as means ± SEM with *P ≤ 0.05, **P < 0.01, and ***P < 0.001. Statistical significance was analyzed via two-way ANOVA followed by Tukey’s multiple comparison *post hoc* test (All *N* = 3).

Given that previous research found that PCN induced SA-βgal through reactive oxygen species (ROS) (18) and to study its role in the proinflammatory response modulated by PA, we measured cytoplasmic ROS production using CellROX staining via flow cytometry (Fig. 6; Fig. S7). Although 24-h PA infection led to a modest increase in ROS, RAPA pretreatment did not enhance or mitigate this effect, indicating that the proinflammatory effects seen with PA and RAPA treatments are ROS-independent (Fig. 6a and b). We also measured the mRNA levels of proinflammatory cytokines and found that RAPA pretreatment with PA infection led to increases in IL-6 and IL-8 (Fig. 6c). To verify downstream proinflammatory immune effects, the levels of ICAM-1 mRNA were measured. ICAM-1 is an epithelial cell-surface glycoprotein that acts as an early T cell activation marker and as an anchor for leukocyte transmigration (42). PA activation of ICAM-1 indicated a robust immune response, and this was further activated by RAPA (Fig. 6d). Cellular cytotoxicity was measured via lactate dehydrogenase (LDH) release into the media after treatment, and PA was found to increase LDH release, as previously shown (30). RAPA pretreatment significantly reduced the amount of LDH released from CFBEs after PA infection but did not reduce the amount of LDH to uninfected levels (Fig. 6e). These results indicate that along with lysosomal homeostasis, PA-induced proinflammatory responses and downstream signaling are also affected by the mTOR axis.

## DISCUSSION

In this study, we found that PA infection of CF bronchial epithelial cells increased SA-βgal activity without increasing several other cell senescence markers, which was validated in ALI and primary cultures. Therefore, we conclude that PA does not induce cell senescence in the CF bronchial epithelium but disturbs lysosomal homeostasis with an increase in SA-βgal activity, an increase in lysosomal pH, and a decrease in lysosomal biogenesis markers. Although PA does not induce cell senescence, it might exert an “aging effect” on CF lysosomes, with SA-βgal being an organelle-specific marker. Rapamycin, a known mTORC1 inhibitor and senolytic, was found to decrease the PA-induced SA-βgal activity and ameliorate the decrease in lysosomal biogenesis, which was independent of both lysosomal neutralization and ROS.

PCN, a virulence factor of PA, increased SA-βgal activity in our study (Fig. 1a), as consistent with previous literature, which was previously interpreted as PCN inducing cell senescence (18, 19, 43). In contrast to these studies, we expanded our study to include the effects of PCN using additional established cell senescence markers and did not find any increase in their levels (Fig. 1b and c). Therefore, we conclude that PCN as one PA-associated virulence factor does not induce cell senescence but affects an aspect of lysosomal homeostasis in the CF bronchial epithelium.

In addition, PA infection itself only led to increased SA-βgal activity without increasing p16, p21, and BCL-xL expression in both CFBEs and 16HBEs (Fig. 2, 3 and 4a through c), despite CFBEs showing an upregulation in these markers due to H2O2 treatment (Fig. 2c). Previous groups found that CFBEs displayed decreased internalization of PA and delayed apoptosis (44), which could imply apoptotic resistance, a feature of cell senescence, but the study did not further investigate other cell senescence markers indicating cell cycle arrest, SA-βgal activity, or inflammation. Two groups found that arginase (45) and Exotoxin T (ExoT) (46) from PA could induce cell cycle arrest in cancer cell culture lines but did not report cellular senescence markers p16 or p21. Only one other group, to our knowledge, has reported the effect of PA infection on cellular senescence marker p16 in a murine sepsis model (cecal ligation and puncture [CLP]), meant to recapitulate a two-hit model of PA secondary infection (47). In this study, researchers found that PA exacerbated an increase in p16 and SA-βgal already seen in their CLP model, which might be needed in our model system by combining an additional proinflammatory stimulus and PA infection.

Since initial data were generated in a CF cell line, we wanted to ensure that our results are translatable to primary cells. We validated our findings by reanalyzing an RNA-seq data set of primary cultures showing that widely published comprehensive cell senescence scores (CellAge and SenMayo) also did not differ between control cells and cells infected with PA (17, 48–50) (Fig. 4d).

Consistent with previous reports, RAPA treatment ameliorated PA-induced SA-βgal activity in CFBEs (Fig. 5a) (35, 36), which was accompanied by a decreased expression of *TFEB* and *LAMP1* (Fig. 5b) (51). While other groups have shown colocalization of PA and LAMP1, indicating that PA could be degraded through lysosomal machinery in macrophages (25, 52), we did not observe this colocalization (Fig. 5c), potentially due to a difference in the phagocytotic capacity of bronchial epithelial cells. Lysosome biogenesis is an important immune function in response to pathogens and injury, and many bacteria have demonstrated the ability to subvert autophagy (24, 53). We showed that RAPA treatment reversed the PA-induced decreases in *TFEB* and *LAMP1* (Fig. 5b and c) and that PA infection reduced RAPA-induced autophagy via LC3BII/I expression (Fig. 5d), consistent with previous reports that PA infection can reduce autophagy (24, 51). Originally a marker for phagocytic cells, CD68 has also been shown to be activated and colocalized with LAMP1 by *E. coli* in epithelial cells to produce a phagocyte-like response (40). Consistent with other groups who have shown that PA can become intracellular and would benefit from downregulating this cellular machinery (24–26), our findings show that PA decreases RAPA-induced *CD68* and that RAPA pretreatment can attenuate this effect (Fig. 5e). Another report indicates PA acting indirectly on the acidification of lysosomes in corneal epithelial cell infection (25), which is consistent with our results (Fig. 5f; Fig. S4). Also consistent with our findings (Fig. 5f), RAPA treatment alone has been shown to be insufficient to acidify lysosomes (54), indicating that PA neutralization of CFBE lysosomes could be independent of mTORC1.

We and others have shown that PA infection can induce ROS production (55, 56) (Fig. 6a and b). Given that we observed only a modest increase in ROS production with PA infection, we cannot rule out that this is due to epithelial cells producing less ROS than neutrophils and macrophages or differences in specific ROS. Several groups have reported different results when measuring proinflammatory cytokines after RAPA treatment (57–60), and we showed that RAPA treatment further increased proinflammatory cytokines IL-1β, IL-8, and IL-6 when combined with PA infection (Fig. 6c). This could be due to cell type-specific signaling and/or a heightened immune response to PA infection due to RAPA restoring lysosomal biogenesis. Other groups have shown that RAPA augments ICAM-1 expression during stress (35, 61), which we show as well (Fig. 6d). RAPA has also previously been shown to reduce LDH expression *in vitro* (62, 63)*,* but this finding had not yet been demonstrated in PA infection or in CFBEs (Fig. 6e). These data indicate that RAPA can affect downstream mediators of the PA-induced proinflammatory response.

Our study has limitations. We completed most of our key signaling experiments in an immortalized cell line, but we verified these results in primary, differentiated bronchial epithelial cells, and lysosome perturbations from PA infection were observed utilizing several markers and methods. Additionally, we validated that our immortalized cell line can express higher levels of senescence markers when stimulated with a known senescence inducer, H_2_O_2_. Due to the highly inflammatory nature of PA infection, *in vitro* infections were only possible up to 24 h without significant cell death. We instead sought to characterize the initial host response to PA infection, with future studies needed to explore chronic and/or recurrent infection models. We also cannot eliminate the possibility that the effects seen with RAPA treatment are not an off-target effect. RAPA is most known for increasing autophagic activity through inhibiting the formation of the mTORC1 complex, but some studies have shown that it can also inhibit the mTORC2 complex necessary for cell proliferation (36). Moreover, although we have identified several key mediators of lysosome dysfunction in response to PA infection, the upstream regulator of these processes remains elusive and should be investigated further in future studies.

**Fig 7:**
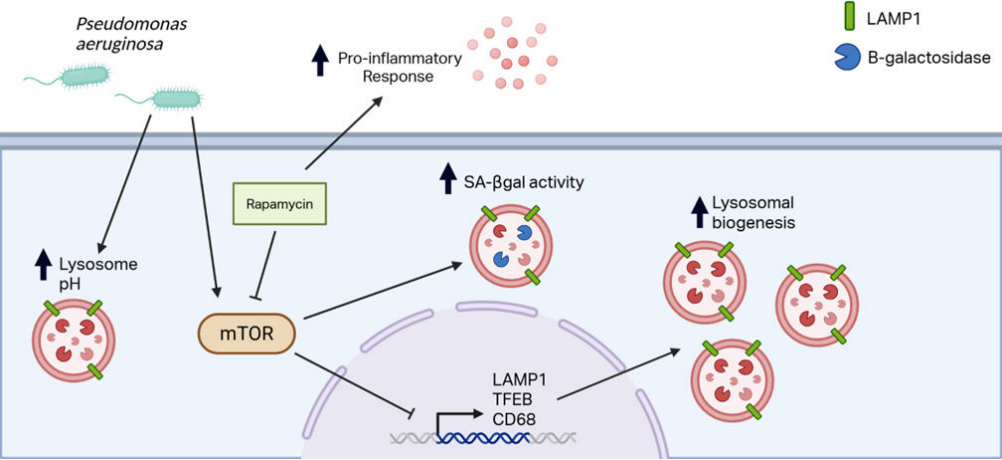
Schematic of how PA infection disrupts lysosomal homeostasis, which can be partially mediated by mTOR inhibitor rapamycin. Created in BioRender.

In summary, we established that PA infection can increase SA-βgal activity without affecting other cellular senescence markers. When we investigated this mechanism further, we found that PA infection also disrupts lysosomal homeostasis in the CF bronchial epithelium through lysosome biogenesis and neutralization, which is partially mediated by RAPA (Fig. 7). These findings suggest that PA infection could contribute to worsened patient outcomes by negatively regulating lysosome function in the lung and contributing to a dysregulated immune response.

Currently, PA infections are often treated with combination antimicrobial therapy (64). However, the ability of PA to become multidrug-resistant and chronically colonize its host is an ever-growing problem, especially in pwCF and other patient populations who are immuno-compromised (2, 65, 66). Given that HEMT is expected to prolong the lives of pwCF but not clear them of infection, chronic PA colonization over an extended period could give rise to higher rates of drug-resistant PA (7, 67). This problem highlights the need for alternative therapeutic strategies, including host-directed treatments that focus on ameliorating PA-induced disruptions. Given this and our findings, further studies are needed to assess the consequences of PA infection on host signaling mechanisms, especially in lysosomal dysfunction and lysosomal senescence as a potential mechanism to target therapeutically.

## MATERIALS AND METHODS

### Cell culture

Immortalized human bronchial epithelial cells (16HBEs) and immortalized human bronchial epithelial cells stably expressing the ΔF508 CFTR mutation via lentiviral vector (CFBEs) were used for experiments and cultured on plates in a medium consisting of Minimum Essential Media (MEM) (Gibco, Billings, MT, USA, 11095-072), supplemented with 10% fetal bovine serum (FBS) (Atlas Biologicals, Fort Collins, CO, USA, FP-0500-A), 1% L-glutamine (Corning, 25-005-CI), 0.5% penicillin/streptomycin (Corning, NY, USA, 30-002-CI), and 0.2% plasmocin (Invivogen, ant-mpp). Cell lines were cultured as previously described (17, 30) at 37°C with 5% CO_2_. In preparation for treatments, cells were seeded onto 12-well plates, with 2.4 × 10^5^ CFBEs or 4.8 × 10^5^ 16 HBE cells added to each well. For experiments performed at the air-liquid interface (ALI), a different type of immortalized human bronchial epithelial cells (CFBE41o-) homozygous for the ΔF508 CFTR mutation and optimized for polarization were cultured and maintained in the same manner as described above. Once cells reached 90% confluency, they were seeded onto Vitrogen Plating Media (VPM)-coated 12 mm Transwell filters (Corning, 3401), grown for 2–4 days with apical media, and then polarized at the air-liquid interface for 10–14 days (30).

### Cell treatments

Treatment with pyocyanin (PCN) (Cayman Chemical, 10009594) was conducted as previously described (18) with 1 µg/mL, 2 µg/mL, and 20 µg/mL PCN diluted in culture media. PCN treatment was initiated at least 24 h after cells were plated and when cells were between 25% and 50% confluency. PCN treatments were conducted for 48 h.

Treatment with hydrogen peroxide (H_2_O_2_) was conducted for 24 h on CFBEs as a previously described positive control of cellular senescence induction (31). H2O2 treatment was initiated at least 24 h after cells were plated and when cells were approximately 50% confluent.

Pretreatment with rapamycin (RAPA) (MedChemExpress, HY-10219) was conducted for 1 h before PA infections and supplemented in the infection media for 24 h. As previously described (68), RAPA was diluted to 100 nM in culture media.

### Preparation of PA and cellular infection

The PA strain PAM57-15 (PAM) was a generous gift from Dr. Susan Birket. Infections with PAM were conducted as previously described (30, 32). Briefly, 1 day prior to bacterial infection, cells were washed with Dulbecco’s phosphate-buffered saline (DPBS) to remove residual antibiotics and replaced with antibiotic-free culture media. PA was cultured in 5 mL of LB broth with shaking overnight at 37°C, 1 day prior to the assay. The overnight cultures of PA were washed twice with MEM +5% L-glutamine without phenol red and diluted to an OD600 of 0.5. Using a 1:5,000 dilution in MEM +5% L-glutamine, an average of inoculum colony-forming units (CFUs) of 1.84 × 10^3^ and multiplicity of infections (MOI) of 0.004 and 0.0002 were established for the CFBE and 16HBE cells, respectively (30). Media containing diluted bacteria were added to at least 90% confluent cells, approximately 5 × 10^5^ CFBE cells or 1 × 10^6^ 16 HBE cells, as determined via light microscopy. The medium was aspirated off the cells after 1 h and centrifuged for 3 min at 8,000 RCF to remove any non-adherent PA. The supernatant was replaced on the apical surface with the addition of 4% L-arginine, as previously described (30, 32). Cells were further co-cultured with PA in 5% CO_2_ at 37°C for an additional 5, 11, or 23 h. At least 75% cell confluence was confirmed before further processing or imaging of cells after treatment.

### Lysosensor pH measurements and imaging

Lysosensor yellow/blue dextran (ThermoFisher, L22460) was used to measure intracellular organelle pH. The Lysosensor is endocytosed and accumulates in endocytic vesicles (endosomes and lysosomes), where it exhibits pH-dependent dual-emission spectra (acidic pH emission = 530 nm; neutral pH emission = 450 nm). The ratio of 530 to 450 nm provides a quantifiable ratio of acidic to neutral pH measurements. A standard curve to correlate known pH measurements to acidic/neutral signal ratios was generated as previously described (69). The acidic/neutral signal ratio (335 nm Abs, 452 nm Em/381 nm Abs, and 521 nm Em) was measured on a microplate reader. For our experiments, cells were cultured and treated as described above, with the addition of the Lysosensor probe (100 uM) into apical media 1 h after PA infection was initiated. After 19–23 h, the medium was removed, cells were washed once with PBS, scraped, and resuspended in 100 uL of PBS. The cell suspension was transferred to a black 96-well optical-bottom microplate (Greiner Bio-One, 655086), centrifuged for 5 min at 300 × *g*, and then measured on a BioTek Synergy Mx microplate reader and analyzed with Gen5 software (BioTek, version 3.08).

To visually confirm the Lysosensor probe, imaging was done on PA-infected CFBEs with Lysosensor. Cells were prepared and treated as described above on chamber-well slides, and imaging was completed using a Nikon Eclipse Ti2 widefield microscope and software Nikon NIS-Elements AR software package (version 5.42.02 Build 1801).

### Protein preparation and western blotting

As previously described (30), all protein lysates were obtained using radioimmunoprecipitation assay buffer with phosphatase inhibitor, phosphatase inhibitor cocktail II (RPI), and protease inhibitor, Roche cOmplete Protease Inhibitor Cocktail (Millipore Sigma). Protein concentrations were determined by Bradford Assay. Thirty micrograms of the protein was loaded in each well. Proteins were separated on 4%–20% precast polyacrylamide gels (BioRad 4561094) and transferred onto 0.2-µm PVDF membranes (Pierce, Thermo Fisher Scientific). As a loading control, one gel was stained for total protein after protein separation using GelCode Blue Stain Reagent (Thermo Scientific) for 1 h. The gel was washed with deionized water overnight and imaged using Amersham Imager 600 system (GE). Membranes were blocked with 5% low-fat milk for 30 min and then incubated overnight with the following primary antibodies: rabbit anti-p21 (Cell signaling 29478), rabbit anti–BCL-xL (Cell signaling 2764S), rabbit anti-p16 (Proteintech 10883-1-AP), and rabbit anti-LC3B-II (Cell signaling 3868) diluted according to the manufacturer’s recommendations. After three washes with TBS with Tween (TBST), membranes were incubated with goat anti-rabbit peroxidase-conjugated antibody (Invitrogen, Thermo Fisher Scientific) at 1:5,000 in 5% low-fat milk for 1 h. After three washes in TBST, the membranes were imaged by chemiluminescence on a ChemiDoc XRS system (Bio-Rad) and acquired using Image Lab software (Bio-Rad). ImageJ (version 1.54g) was used to measure the densitometry of positive signals on the membranes. These data were then normalized by the total protein from the GelCode Blue Stain Reagent image.

### RNA isolation and RT-qPCR

Total RNA was isolated from cells using the GeneJET RNA Purification Kit (ThermoScientific) as described by the manufacturer (17). RNA concentration for each sample was assessed using a NanoDrop, and cDNA was synthesized using a Maxima H Minus cDNA Synthesis Master Mix with the dsDNase kit (ThermoFisher). RT-qPCR was performed on an Applied Biosystems StepOnePlus using TaqMan primers interleukin-8 (Hs00174103_m1, CXCL8), interleukin-6 (Hs00174131_m1, IL-6), interleukin 1-β (Hs01555410_m1, IL-1β), cyclin-dependent kinase inhibitor 1A (referred to as p21 in this manuscript) (Hs00355782_m1), cyclin-dependent kinase inhibitor 2A (referred to as p16 in this manuscript) (Hs00923894_m1), lysosomal-associated membrane protein 1 (Hs00174766_m1), transcription factor EB (Hs00292981_m1), CD68 (Hs00154355_m1), intracellular adhesion molecule 1 (Hs00164932_m1), and reference gene glyceraldehyde 3-phosphate dehydrogenase (GAPDH). Fold change was calculated as previously described (31); in brief, average CT values were normalized to GAPDH and then to control samples, resulting in fold change using the following formula: POWER(2, -ΔΔCT).

### SA-βgal staining

Immediately following treatment, cytochemical staining for SA-βgal was performed using a senescence-associated β-galactosidase staining kit (Cell Signaling Technologies 9860) following the protocol provided. To quantify the extent of β-galactosidase staining, we captured four images in different regions of each cell culture plate well and three different wells as technical replicates and counted all cells and all β-galactosidase-positive cells from the nine images and counted the ratio of β-galactosidase-positive cells to total cells. Experiments were done in biological triplicate. Images were taken on a Nikon Eclipse Ts2 at 20× magnification (scale bar = 100 µm) using NIS-Elements D (version 5.30.05) and quantified using ImageJ (version 1.54g).

### RNA sequencing transcriptomic data

These data shown here have been deposited in NCBI’s Gene Expression Omnibus (70) and are accessible through GEO Series accession number GSE268718. The published data set was re-analyzed for senescence and proinflammatory markers of interest. Senescence scores were generated from published gene sets associated with aging and senescence, as described previously (17, 33, 34). The Gene Set Variation Analysis (GSVA) package was used to compare the published data set to the generated senescence scores using R (version 4.3.1) (71).

### Immunofluorescence staining and imaging

CFBE cells were grown and treated with RAPA and PA strain PAM57-15 as previously described on 8-well chamber slides. Immediately following treatment, cells were washed once with PBS and fixed using 4% paraformaldehyde for 10 min. The cell membranes were permeabilized using 0.1% Triton, blocked with 5% BSA in PBS, and incubated for 2–3 h in a 1:1,000 dilution of mouse anti-LAMP1 antibody (H4A3; obtained from the Developmental Studies Hybridoma Bank, created by the NICHD of the NIH and maintained at The University of Iowa, Department of Biology, Iowa City, IA 52242). The cells were then incubated with chicken anti-mouse IgG (H + L) cross-adsorbed secondary antibody, Alexa Fluor 488 for 1 h, washed with PBS, and mounted using ProLong Gold Antifade Mountant.

Imaging and processing were done using a Zeiss Axio Imager.Z2, Zeiss LSM 800 camera, and ZEN Blue (version 2.6) taken using the Airyscan mode at 40× oil objective and 60× digital zoom.

### Reactive oxygen species staining and flow cytometry

Cells were infected with PA as previously described for 20 h. The infection medium was removed from cells; fresh uninfected medium was added with 0.2% CellROX Deep Red Reagent (Invitrogen C10422) to assess reactive oxygen species production and incubated at 37°C for 30 min. Wells were washed once with 1× PBS, incubated with trypsin at 37°C for 8 min, then pelleted, and finally washed with ice-cold PBS + EDTA (2.5 mM). Cells were resuspended and then incubated with 1:10 diluted live-dead stain Zombie Aqua (BioLegend 423102) in PBS + EDTA on ice for 10 min in the dark. Cells were spun at 800 × *g* for 10 min at 4°C, washed with PBS + EDTA, and then fixed with 2% PFA for 20 min on ice. Cells were pelleted and resuspended in 1 mL of PBS + EDTA for storage at 4°C in the dark. Within 7 days of staining, cells were pelleted, resuspended in 300 uL of PBS + EDTA, and then filtered using 5 mL polystyrene tubes with cell-strainer cap (Falcon 352235).

Flow cytometry was done using a BD FACSymphony A3 instrument with violet laser 405 nm and detector BV510 for Zombie Aqua and red laser 640 nm and detector APC for CellRox staining. Data collection and initial gating were done using BD FACSDiva software (version 8.0.1). Further analysis was completed using FlowJo (versions 10.10.0 and 11.0.2).

### Lactate dehydrogenase assay

Cellular cytotoxicity was measured via lactate dehydrogenase (LDH) released into the media (30, 72). LDH release was quantified using a Lactate Dehydrogenase Assay Kit (Sigma-Aldrich MAK464) according to the manufacturer’s directions.

### Statistics

Data were analyzed with GraphPad Prism 10 (version 10.6.1) as previously described (17, 30) using unpaired two-tailed Student’s t test, one-way ANOVA, or two-way ANOVA for a minimum of three independent experiments. Data are shown with individual values from each experiment ±SEM. Statistical significance was accepted at a *P* value of less than 0.05. Trending significance was accepted at a *P* value of less than 0.2.

## Data Availability

The RNA-seq data analyzed in this manuscript are accessible through GEO Series accession number GSE268718 (70). The other data generated or analyzed during this study are available from the corresponding author upon reasonable request.

## SUPPLEMENTAL MATERIAL

Supplemental figures iai.00347-26-s0001.pdf Fig. S1 to S7.

ASM does not own the copyrights to Supplemental Material that may be linked to, or accessed through, an article. The authors have granted ASM a non-exclusive, world-wide license to publish the Supplemental Material files. Please contact the corresponding author directly for reuse.
