# Supplementary material for: Pseudomonas aeruginosa infection causes lysosomal dysfunction in the cystic fibrosis bronchial epithelium
Source: Infect Immun. 2026 Jun 30;94(8):e00347-26. doi: 10.1128/iai.00347-26 (PMC13459585; doi:10.1128/iai.00347-26)
Supplement: Supplementary file 1 [file iai.00347-26-s0001.pdf]

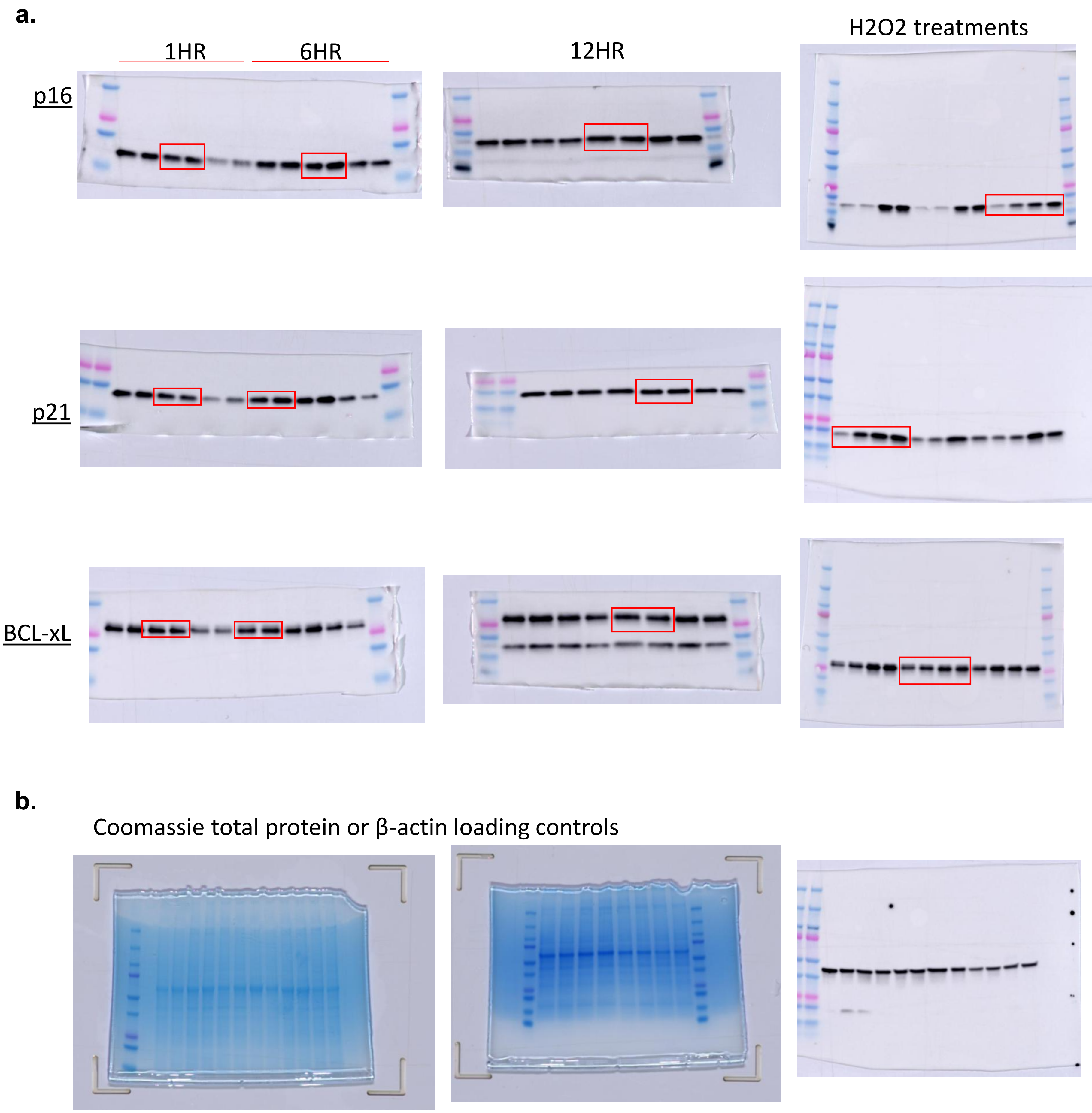

Supplementary Figure S1. Uncropped immunoblot images from Fig. 2. **A)** For the manuscript, a horizontal region was cropped for the bands of interest indicated by a red box on each picture. **B)** Western blot quantification was normalized for Fig. 2B) total protein using Coomassie staining or Fig. 2C) β-actin. No additional modifications were performed.

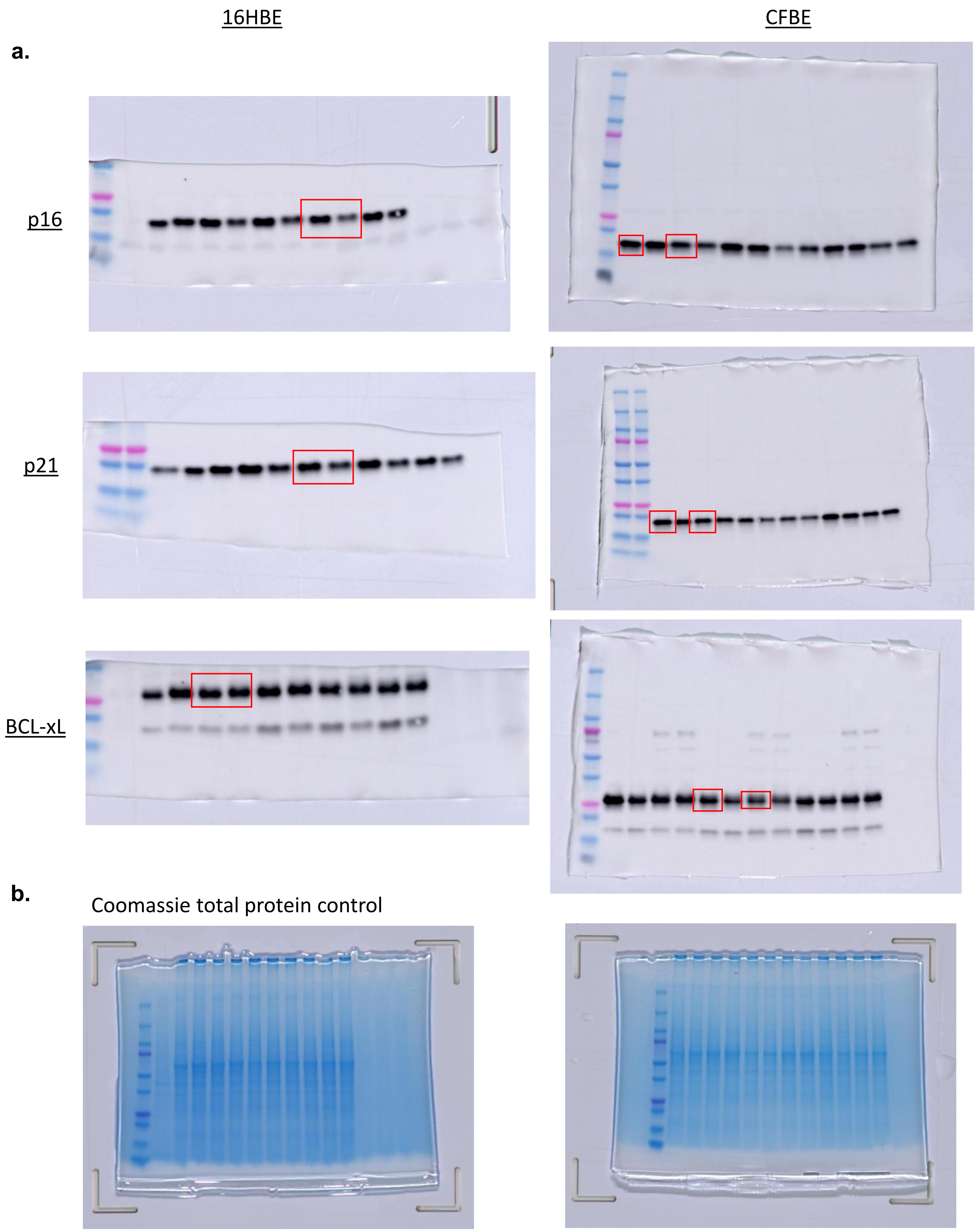

Supplementary Figure S2. Uncropped immunoblot images from Fig. 3B. **A)** For the manuscript, a horizontal region was cropped for the bands of interest indicated by a red box on each picture. **B)** Western blot quantification was normalized to total protein using Coomassie staining. No additional modifications were performed.

a.

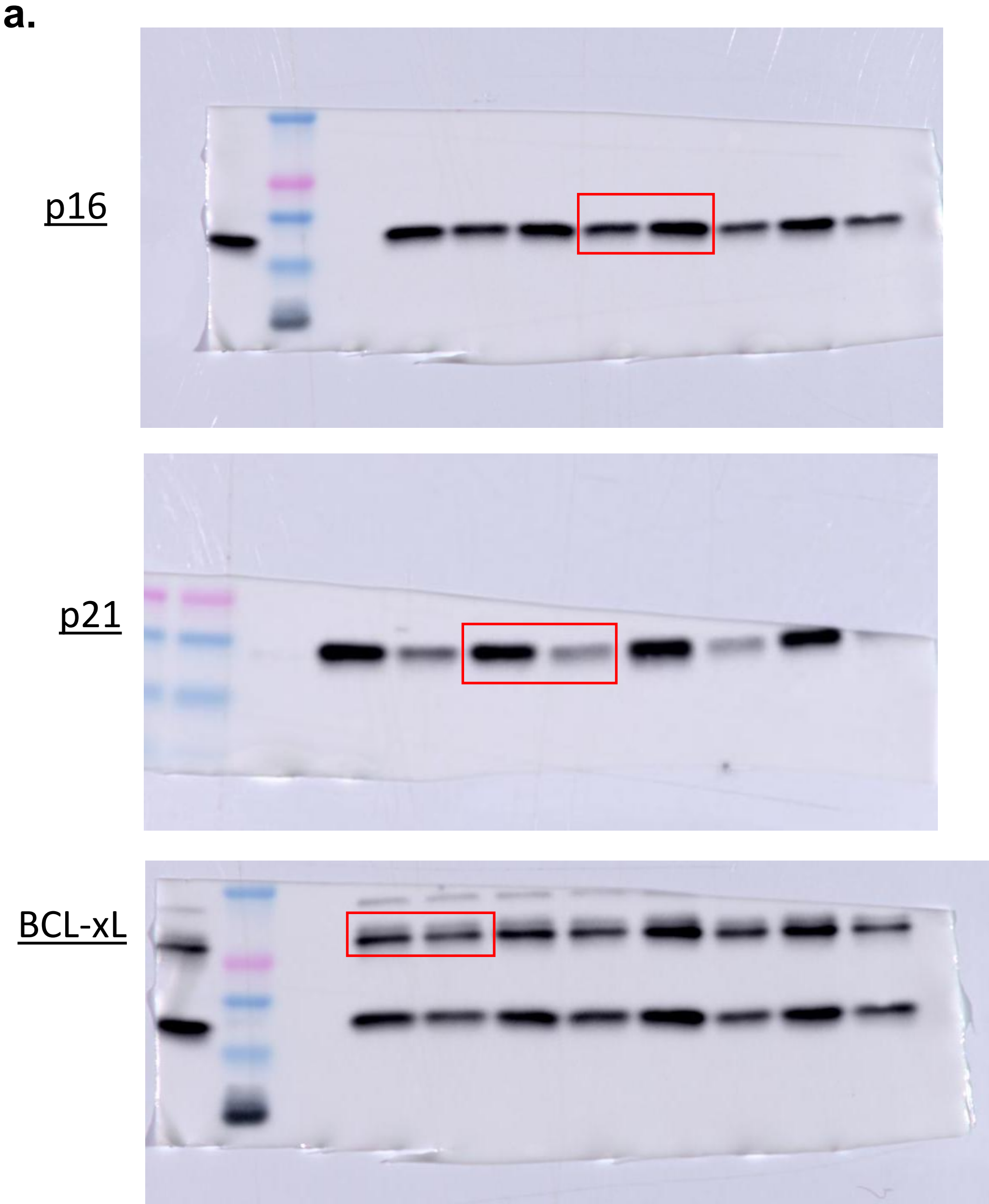

b.

Coomassie total protein control

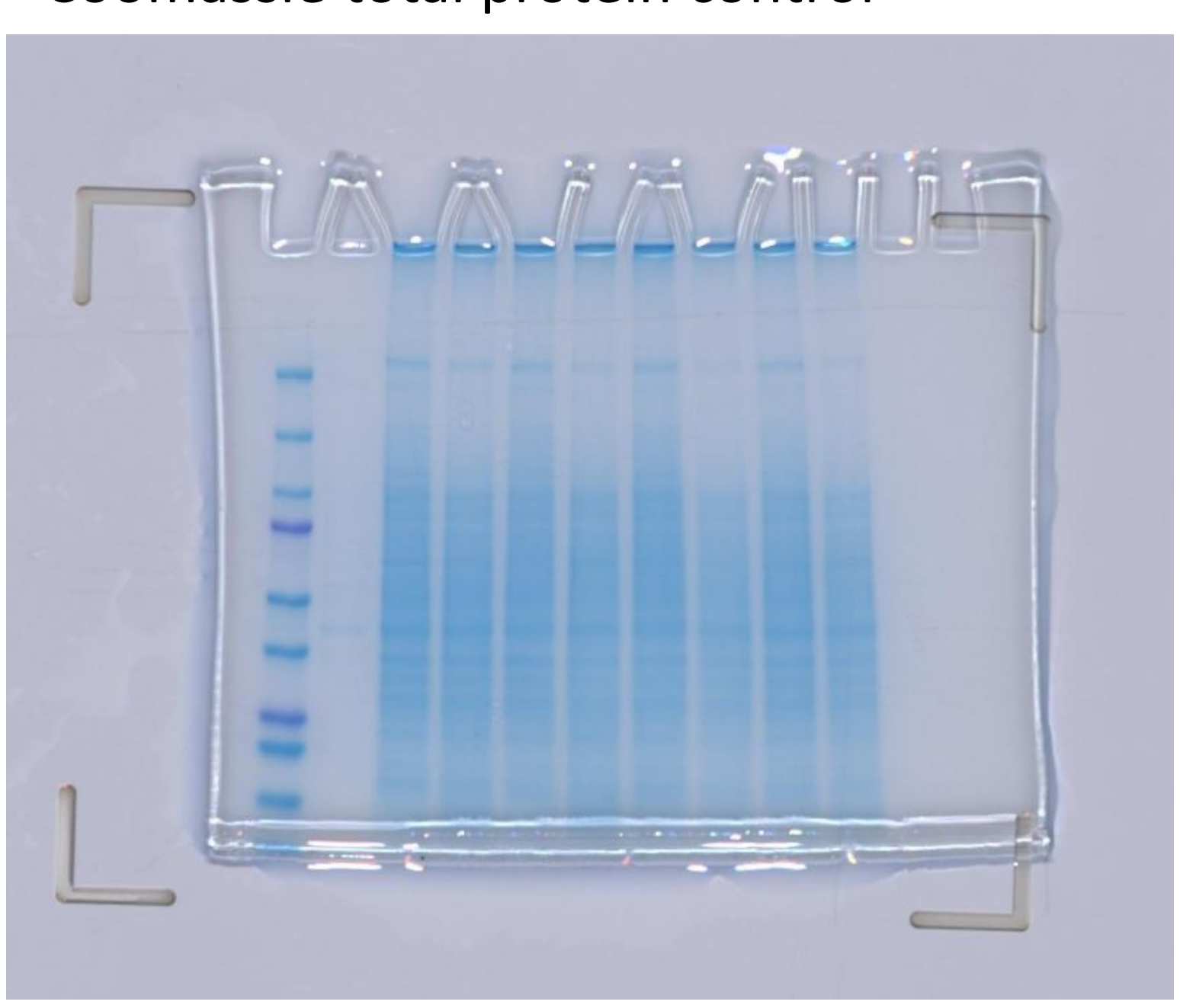

Supplementary Figure S3. Uncropped immunoblot images from Fig. 4A. **A)** For the manuscript, a horizontal region was cropped for the bands of interest indicated by a red box on each picture. **B)** Western blot quantification was normalized to total protein using Coomassie staining. No additional modifications were performed.

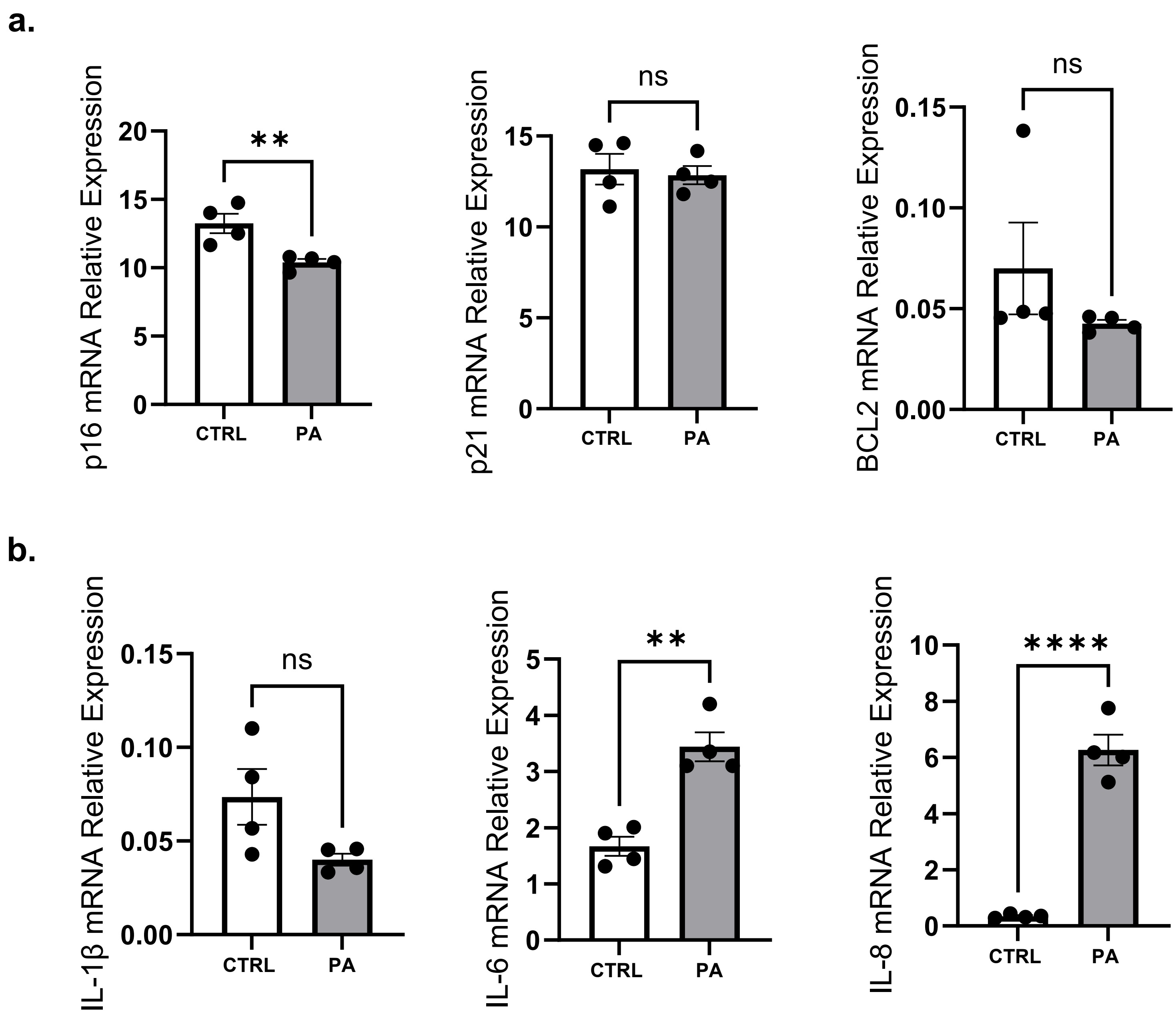

Supplementary Figure S4. A) Relative mRNA expression of *p16, p21*, *BCL2,* **B)** *IL-1β, IL-6* and *IL-8* after a 24-hour PA infection in CFBE41o- cultured at the air-liquid interface.

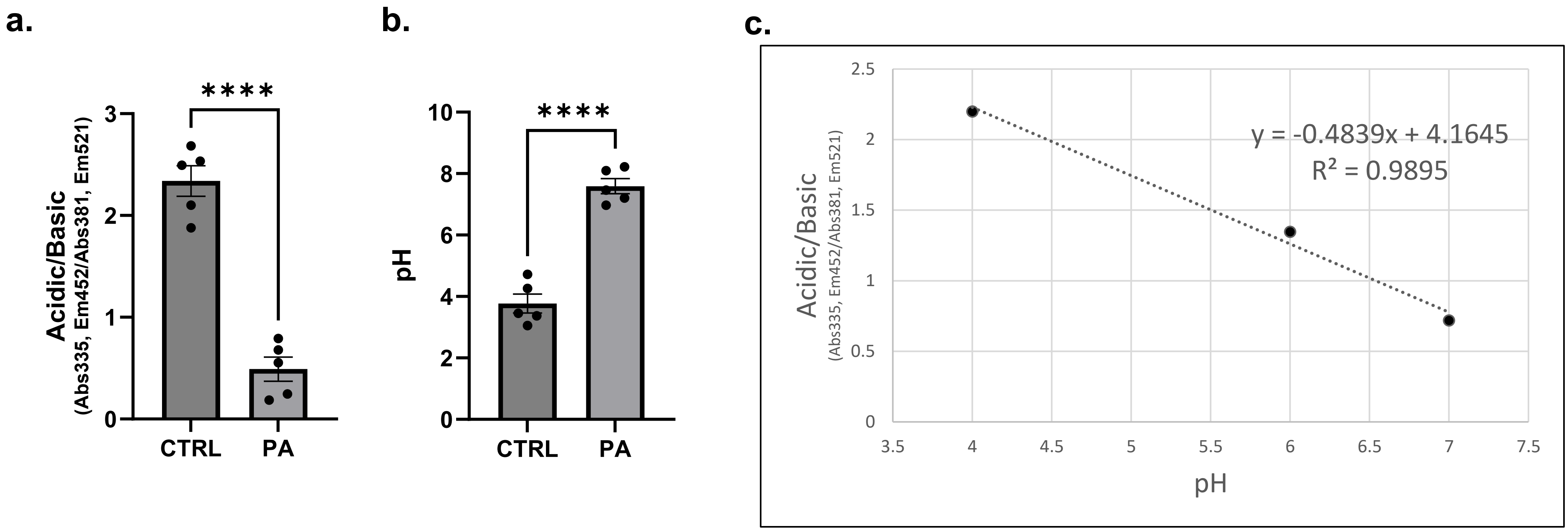

Supplementary Figure S5. PA infection effect on lysosomal pH. **A)** Acidic/basic signal ratio generated from fluorescent readings at Abs335, Em452/Abs381, Em521 in CFBEs after 24-hour PA infection. **B)** pH measurement generated from Acidic/basic ratio in panel A and **C)** calibration curve of CFBEs treated with known pHs. All values are mean ± SEM with $*p \leq 0.05$, $**p < 0.01$, and $***p < 0.001$. Statistical significance was analyzed via unpaired Student's t test (N=5).

a.

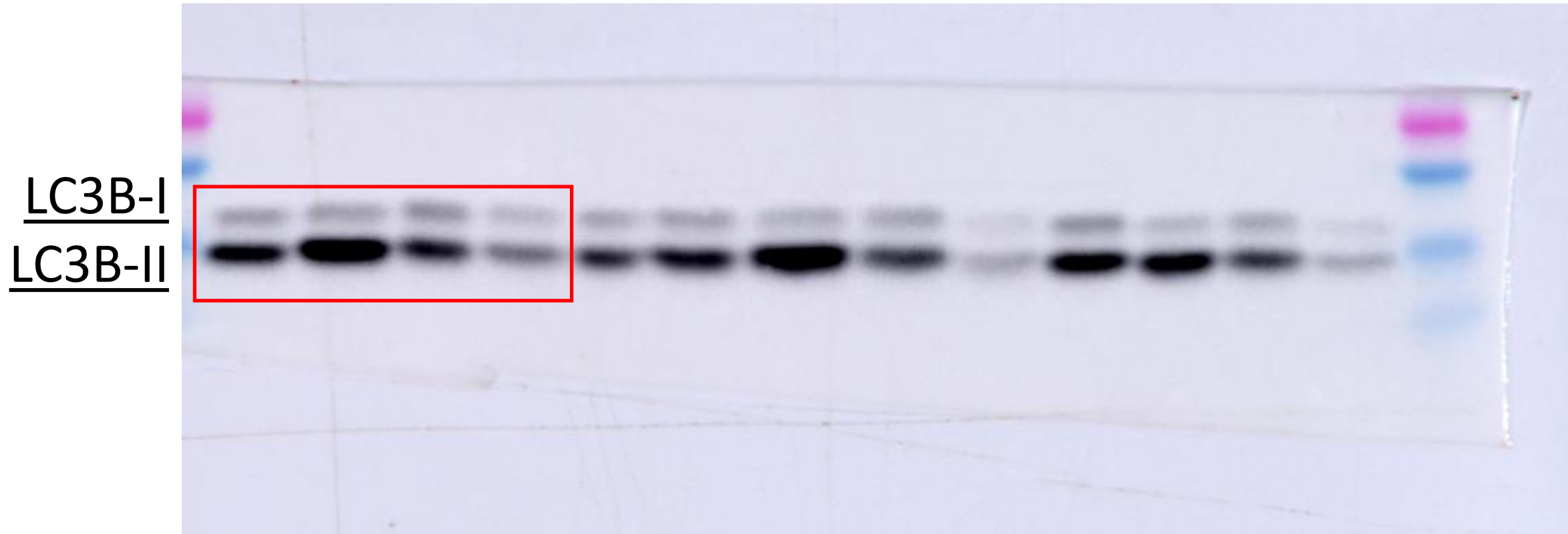

b.

Coomassie total protein control

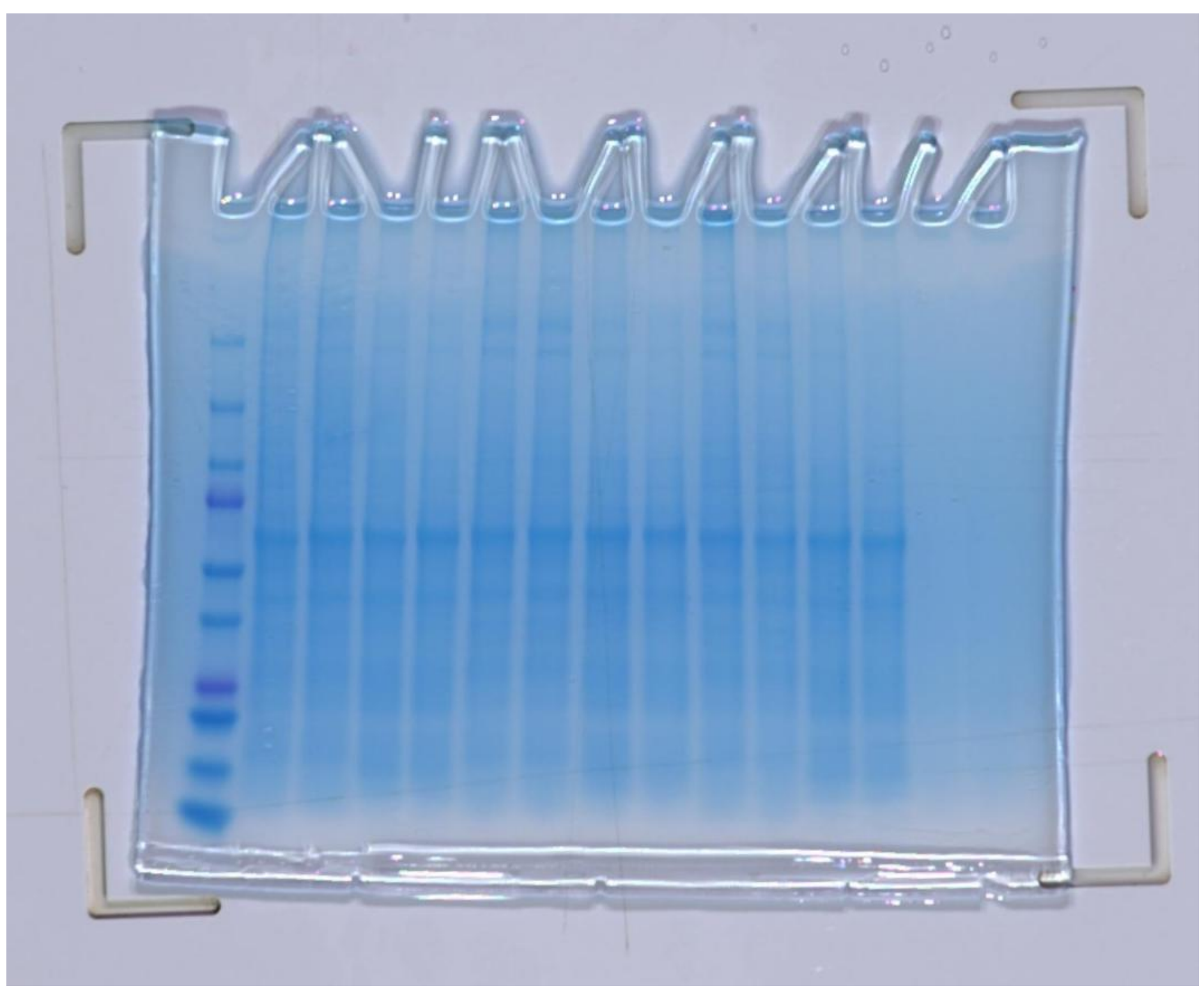

Supplementary Figure S6. Uncropped immunoblot images from Fig. 5D. **A)** For the manuscript, a horizontal region was cropped for the bands of interest indicated by a red box on each picture. **B)** Western blot quantification was normalized to total protein using Coomassie staining. No additional modifications were performed.

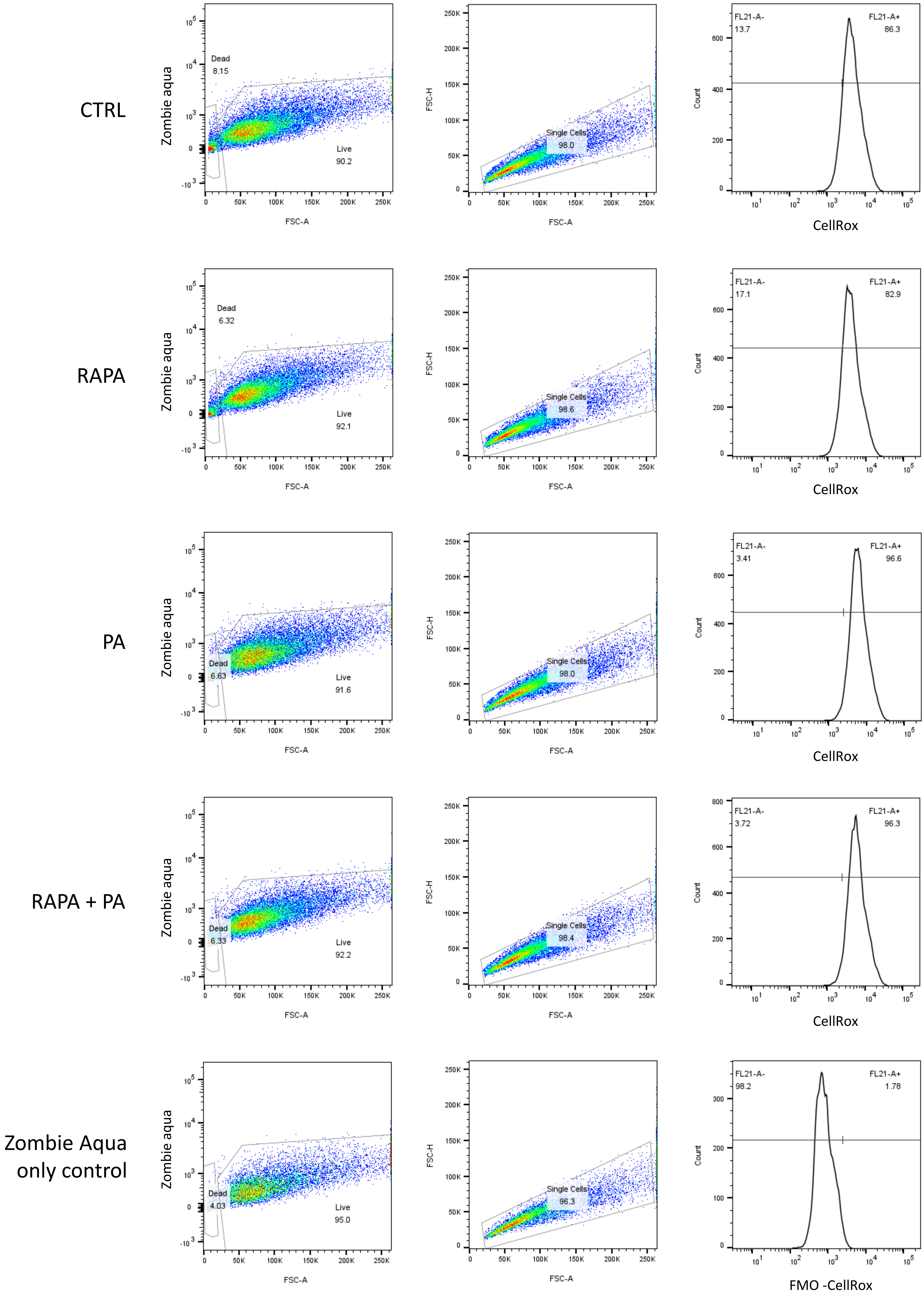

Supplementary Figure S7. Flow cytometry gating strategies.
